# Critical parameters maintaining authentic CRAC channel hallmarks

**DOI:** 10.1007/s00249-019-01355-6

**Published:** 2019-03-21

**Authors:** Adéla Krizova, Lena Maltan, Isabella Derler

**Affiliations:** Institute of Biophysics, Johannes Kepler University of Linz, Gruberstrasse 40, 4020 Linz, Austria

**Keywords:** Calcium, CRAC channel, STIM1, Orai1, STIM–Orai interaction, Orai gating, Gain-of-function mutants, Electrophysiology, FRET, Structural resolution

## Abstract

Ca^2+^ ions represent versatile second messengers that regulate a huge diversity of processes throughout the cell’s life. One prominent Ca^2+^ entry pathway into the cell is the Ca^2+^ release-activated Ca^2+^ (CRAC) ion channel. It is fully reconstituted by the two molecular key players: the stromal interaction molecule (STIM1) and Orai. STIM1 is a Ca^2+^ sensor located in the membrane of the endoplasmic reticulum, and Orai, a highly Ca^2+^ selective ion channel embedded in the plasma membrane. Ca^2+^ store-depletion leads initially to the activation of STIM1 which subsequently activates Orai channels via direct binding. Authentic CRAC channel hallmarks and biophysical characteristics include high Ca^2+^ selectivity with a reversal potential in the range of + 50 mV, small unitary conductance, fast Ca^2+^-dependent inactivation and enhancements in currents upon the switch from a Na^+^-containing divalent-free to a Ca^2+^-containing solution. This review provides an overview on the critical determinants and structures within the STIM1 and Orai proteins that establish these prominent CRAC channel characteristics.

**Electronic supplementary material:**

The online version of this article (10.1007/s00249-019-01355-6) contains supplementary material, which is available to authorized users.

## Introduction

Ca^2+^ is one main key player that controls signalling pathways in the human body. It serves as a second messenger transducing signals in various cell types and is responsible for processes such as exocytosis, gene transcription, cell motility and apoptosis. One of the most prominent Ca^2+^ entry pathways into the cell is through the Ca^2+^ release-activated Ca^2+^ (CRAC) ion channel. It is activated upon Ca^2+^ store-depletion of the endoplasmic reticulum (ER) which is triggered by the binding of inositol 1,4,5-trisphosphate (IP_3_) to IP_3_-receptors located in the ER membrane (Berridge et al. 2003; Parekh and Putney 2005). These Ca^2+^ currents are mediated by two proteins, the Ca^2+^-sensing stromal interaction molecule 1 (STIM1) and the plasma membrane-spanning Orai1 protein (Feske et al. 2006; Liou et al. 2005; Roos et al. 2005; Zhang et al. 2005). They are sufficient to fully reconstitute CRAC channels and their unique biophysical properties (Gudlur et al. 2014).

Proper communication of STIM and Orai proteins is indispensable for unrestricted functions of healthy cells. Several gain- (Morin et al. 2014) or loss-of-function (Feske et al. 2006; Thompson et al. 2009) mutations (Table 1) have been described to lead to drastic defects in cell signalling pathways (Berna-Erro et al. 2012) and can be the reason for diseases like severe combined immune deficiency (SCID), Stormorken syndrome (Feske et al. 2006) and tubular aggregate myopathy (Bohm et al. 2017; Lacruz and Feske 2015; Nesin et al. 2014).

**Table 1:** List of Orai mutants differentiated by their activation properties

	Store operated	Gain of function	Loss of function	References
Orai1 wildtype	X			
Orai1 L74I	X			Derler et al. (2016b)
Orai1 Y80S	X			Derler et al. (2016b)
Orai1 L74/W76E/R/S	X			Derler et al. (2013a)
Orai1 K85E			X	Lis et al. (2010)
Orai1 R91W			X	Feske et al. (2006)
Orai1 S97C	X			Garibaldi et al. (2017)
Orai1 G98C/D/P	X			Yamashita et al. (2017), Zhang et al. (2011)
Orai1 G98R			X	Lian et al. (2017)
Orai1 G98S		X		Bohm et al. (2017)
Orai1 F99C/G/M/S/T/Y/W		X		Yamashita et al. (2017)
Orai1 V102A/C/G/S/T		X		McNally et al. (2012)
Orai1 V102I/L/M/V	X			McNally et al. (2012)
Orai1 A103E			X	McCarl et al. (2009)
Orai1 E106Q			X	Vig et al. (2006)
Orai1 V107M	X			Bohm et al. (2017)
Orai1 H134S/A/C/T/V/Q/E/M		X		Frischauf et al. (2017), Yeung et al. (2018)
Orai1 H134K/W			X	Yeung et al. (2018)
Orai1 A137V		X		Frischauf et al. (2017)
Orai1 L138F		X		Endo et al. (2015)
Orai1 M139V		X		Frischauf et al. (2017)
Orai1 S141C		X		Yeung et al. (2018)
Orai1 S159L		X		Frischauf et al. (2017)
Orai1 L174D			X	Zhou et al. (2016)
Orai1 W176C		X		Srikanth et al. (2011)
Orai1 A177D		X		Frischauf et al. (2017)
Orai1 V181A			X	Derler et al. (2018)
Orai1 G183A			X	Srikanth et al. (2011)
Orai1 G183D			X	Frischauf et al. (2017)
Orai1 T184M	X			Bohm et al. (2017), Bulla (2018)
Orai1 L185A		X		Fahrner et al. (2018a)
Orai1 F187C		X		Yeung et al. (2018)
Orai1 E190C		X		Yeung et al. (2018)
Orai1 E190Q	X			Prakriya et al. (2006)
Orai1 L194P			X	Lian et al. (2017), McCarl et al. (2009)
Orai1 A235C		X		Yeung et al. (2018)
Orai1 S239C		X		Yeung et al. (2018)
Orai1 G247S		X		Frischauf et al. (2017)
Orai1 F250C		X		Yeung et al. (2018)
Orai1 P245L		X		Nesin et al. (2014)
Orai1 L273S			X	Muik et al. (2008)
Orai1 L273D			X	Li et al. (2011)
Orai1 L276D			X	Navarro-Borelly et al. (2008)

STIM1 functions as a calcium sensor within the ER membrane (Liou et al. 2005; Roos et al. 2005; Zhang et al. 2005), while Orai1 acts as the pore unit within the plasma membrane (PM) of the cell (Prakriya et al. 2006; Vig et al. 2006). Together, they form the CRAC channel. Upon ER Ca^2+^ store-depletion, STIM1 proteins lose the bound Ca^2+^, form oligomers within the ER–PM junctions and capture an active conformation (Liou et al. 2005). STIM1 binding to Orai1 leads to the activation of this Ca^2+^ ion channel, likely due to a global rearrangement of the whole channel complex (Derler et al. 2016a; Palty et al. 2015).

The understanding of the two molecular key players of the CRAC channel has been strongly enhanced by structural resolution studies. Regarding STIM1, only N- and C-terminal fragments have so far been resolved at the structural level (Novello et al. 2018; Stathopulos et al. 2006, 2008, 2013; Yang et al. 2012), while a structure of full-length STIM1 is currently missing. A major step in the CRAC channel field represents the structural resolution of *Drosophila melanogaster* Orai (Hou et al. 2012, 2018) and related mutants. These structures have revealed detailed insight in the inter- and intramolecular interactions of the STIM1 and Orai1 proteins. Furthermore, they provide a basis for a better understanding of their unique activation mechanisms and associated conformational changes.

### STIM1, a Ca^2+^-sensing protein located in the ER

The STIM family includes two homologous single-pass transmembrane proteins, STIM1 and STIM2 (Liou et al. 2005; Soboloff et al. 2006; Williams et al. 2001). Sequence alignment of these two proteins reveals a ~ 61% identity (Soboloff et al. 2012). They are expressed mainly in the membrane of the ER as part of CRAC channels (Manji et al. 2000). While STIM1 senses drastic changes in ER Ca^2+^ levels (*K*_D_ = 200 µM), STIM2 gets activated already upon small changes of luminal Ca^2+^ concentrations (*K*_D_ = 500 µM) (Brandman et al. 2007). Throughout this review, we will focus on the role of STIM1.

**Fig. 1:**
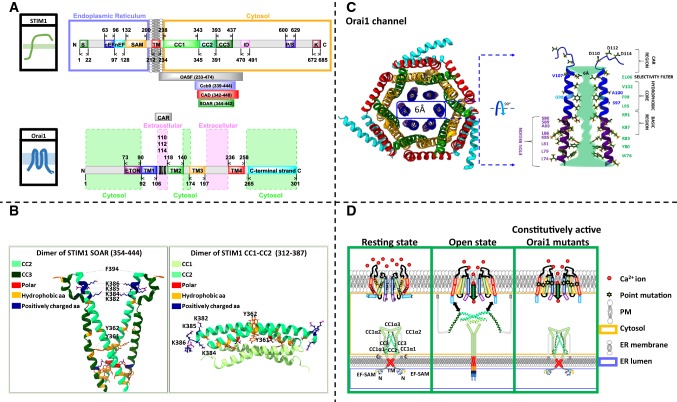
Basic features on STIM1 and Orai1. **A** Scheme showing the critical regions within full-length human STIM1 and Orai1 that regulate their interplay. Important STIM1 fragments, as OASF, Ccb9, CAD, and SOAR are further represented as insets. **B** The X-ray structure of STIM1 SOAR dimer (left) and the NMR structure consisting of a dimer of STIM1 CC1_α3_–CC2 monomers (right). STIM1 SOAR dimers form a V-shape and consist of CC2 and CC3 domains. Each monomer resembles the capital letter “R”. Essential residues that mediate STIM1–STIM1 or STIM1–Orai1 interactions and that are described in the review are highlighted. The STIM1 NMR structure shows that STIM1 CC1_α3_–CC2 monomers couple in an antiparallel manner. Each monomer exhibits a sharp kink between the two coiled–coil domains. **C** The scheme shows the hexameric assembly of human Orai1 according to the *Drosophila* dOrai crystallography structure. The inner ring surrounding the pore is formed by TM1, while the other TM domains within the 6 subunits are arranged into two concentric rings around the pore (left). The cartoon shows two opposite TM1 domains with the cytosolic and extracellular helical extension and represents the important residues lining the pore. The pore can be separated into the CAR region, the selectivity filter, the hydrophobic core, the basis region and the ETON region. **D** The scheme shows STIM1 and Orai1 in the resting state, how Orai channel activation occurs via STIM1 and how Orai channel becomes constitutively active upon single-point mutation independent of STIM1

Starting at the N-terminus, STIM1 contains an ER signal peptide that indicates the location within the ER as its deletion leads to loss of localization in the ER (Hewavitharana et al. 2007; Nilsson et al. 2015). Next, a canonical and a non-canonical EF-hand motif followed by a sterile α-motif (SAM) are acting together as a Ca^2+^ sensor (Stathopulos et al. 2006). Here, the canonical EF-hand can bind Ca^2+^ whereas the non-canonical EF-hand stabilizes the canonical one (Payandeh et al. 2011; Stathopulos et al. 2008) (Fig. 1A).

The EF-SAM domain is followed by the transmembrane region spanning from the luminal to the cytosolic side of the STIM protein. In the dimeric state of STIM1, the two 20 amino acid-long STIM1 TM regions cross each other with an angle of 45° (Ma et al. 2015).

The STIM1 C-terminus is divided into three coiled–coil domains (CC1-3) followed by a more flexible region. Small STIM1 C-terminal fragments [OASF (aa 233–474), SOAR (aa 344–442), CAD (aa 342–448) or Ccb9 (aa 339–444)] have been elucidated to be sufficient for Orai channel activation (Kawasaki et al. 2009; Muik et al. 2009; Park et al. 2009; Yuan et al. 2009) (Fig. 1B). Structural resolution of STIM1 C-terminal fragments (SOAR-like fragment: aa 354–444, SOAR overlapping fragment: aa 312–387), both including CC2 and CC3, has enabled to understand key interaction sites within and between STIM1 monomers. Both structures exhibit that the STIM1 fragments form dimers in an antiparallel manner, with a crossing point at residue Y361 (Stathopulos et al. 2013; Yang et al. 2012). Nevertheless, their overall structures are largely distinct and a structure of full-length STIM1 is still missing. Hence, additional structural studies are still required to clearly define the conformation of STIM1 in the closed and open state.

The STIM1 CC3 region is followed by an inhibitory region or CRAC modulatory domain (CMD) which is critical in adjusting fast Ca^2+^-dependent inactivation (FCDI) (Muik et al. 2009). A proline–serine-rich and a lysine-rich region contribute to the targeting of STIM1 to the plasma membrane (Li et al. 2007) (Fig. 1B) and complete the architectural features of STIM1.

### The Orai channels

The Orai family includes three members, Orai1-3, which form as a second part the CRAC channel complex, the pore unit in the plasma membrane (Vig et al. 2006). While the structure of human Orai (hOrai) remains elusive, in 2012 Hou et al. (Hou et al. 2012) discovered the crystal structure of *Drosophila melanogaster* Orai (dOrai), that reveals a hexameric complex (Fig. 1C). Meanwhile, also the structure of two dOrai mutants is available, whereby one represents an open and the other one a closed state (Hou et al. 2018).

Each Orai subunit consists of four transmembrane domains (TM) connected by flexible loops, two facing into the extracellular space, while one is pointing into the cytosol (Feske et al. 2006). Both, N- and C-termini are cytosolic strands. The pore is formed by the six TM1 regions (Hou et al. 2012). It is surrounded by the other TM domains in two ring-like structures, whereas one is formed by TM2/3 and the second by TM4. TM4 includes a kink at the proline P288 in dOrai (P245 in hOrai1), thus dividing it into two parts. The C-terminus is connected to TM4 by a bent region (Hou et al. 2012) the so-called nexus (Zhou et al. 2016) (Fig. 1C). Interestingly, the C-termini of two neighbouring Orai subunits arrange in an antiparallel manner. Hence, the hexameric Orai complex can be also seen as a trimer of three Orai dimers. A comparison of the dOrai structure published in 2012 (Hou et al. 2012), that is assumed to capture the closed state, with the novel crystal structure of dOrai H206A, potentially exhibiting the open state, revealed the following structural differences (Hou et al. 2018). TM4 together with the C-terminus is straightened (Hou et al. 2018) and the diameter of the pore in the basic region is dramatically enlarged (Hou et al. 2018), suggesting that these structural alterations are critical for Orai pore opening.

The pore can be roughly divided into four parts (Fig. 1C). Coming from the outside of the cell, a Ca^2+^-accumulating region (CAR) (Frischauf et al. 2015), consisting of three negatively charged amino acids (D110, D112, and D114) of each of the six Orai1 subunits, contributes to the attraction of Ca^2+^ ions. It is followed by the selectivity filter that is formed by a ring of six glutamates (E106) (Hou et al. 2012; Prakriya et al. 2006) at which the pore displays a diameter of ~ 3.8 Å (Yamashita et al. 2007). The selectivity filter is followed by a hydrophobic segment that includes the hydrophobic residues: L95, F99 and V102 (Hou et al. 2012). At the cytosolic side, the Orai pore contains a basic region constituted by the extended transmembrane Orai1 NH_2_-terminal (ETON) region (Derler et al. 2013a). It is also part of the N-terminus and forms a cytosolic helical extension of TM1 (Derler et al. 2013a). Positively charged residues (R83, K85, R91) in the ETON region are lining the pore and may hinder the Ca^2+^ ion flow while Orai1 is in a resting/closed state (Hou et al. 2012) (Fig. 1C).

### Activation of STIM1

Before STIM1 gets activated upon store depletion, it shows based on fluorescence microscopy a more homogeneous distribution in the ER (Baba et al. 2006; Luik et al. 2006; Zhang et al. 2005). Upon store depletion, fluorescence-labelled STIM1 proteins move into punctate structures, suggesting that they oligomerize. This resting localization is ensured by (i) Ca^2+^ bound to the STIM1 EF-SAM domain via a negatively charged binding site (Stathopulos et al. 2008), (ii) STIM1 TM domains that cross at a certain angle (Ma et al. 2015), and (iii) an intramolecular clamp within STIM1 C-terminus (Fahrner et al. 2014; Korzeniowski et al. 2010) (Fig. 1D).

Ad (i) store-depletion leads to the dissociation of the Ca^2+^ ion from the canonical EF-hand and triggers a conformational change of the EF-SAM domain. Exposure of hydrophobic areas of the EF-SAM domain induces oligomerization of nearby EF-SAM domains, which is in line with punctate localization of the active STIM1 proteins in the plasma membrane (Stathopulos et al. 2006, 2008; Zheng et al. 2011) (Fig. 1D).

Ad (ii) store-depletion induced conformational changes in the EF-SAM domain provide an activation signal that propagates via the STIM1 TM domain towards the C-terminus, thus resulting in Orai activation (Fahrner et al. 2018b; Ma et al. 2015; Stathopulos et al. 2006, 2008). Here, the TM domain has been proposed to undergo a conformational change leading to a decrease in their crossing angle (Ma et al. 2015) (Fig. 1D).

Ad (iii) further in the C-terminus, an intramolecular clamp between CC1 and CC3, manifesting the closed state, is released upon store depletion leading to the exposure of SOAR (Fahrner et al. 2018b) which is an essential binding region for ORAI1 (Frischauf et al. 2011) (Fig. 1D).

In addition, STIM1 oligomerization is supported by accessory proteins that are already localized at the ER–PM junctions, like the microtubule tip attachment protein EB1, the extended synaptotagmin family, ESyt1 and junctate (Chang et al. 2013; Giordano et al. 2013; Grigoriev et al. 2008; Srikanth et al. 2012; Treves et al. 2004, 2010).

### Activation of the CRAC channel complex via STIM1–Orai1 coupling

Upon activation of STIM1, it couples to and activates the Orai1 channel, which leads to Ca^2+^ influx into the cell. Essential coupling sites and how the pore opening might take place are described in the following sections.

#### Essential coupling domains within STIM1

The cytosolic C-terminus of STIM1 includes the most prominent site for direct coupling to Orai1. It has been identified by minimal C-terminal fragments [OASF (aa 233–474), SOAR (aa 344–442), CAD (aa 342–448) or Ccb9 (aa 339–444)] that are sufficient to initiate Orai1 activation (Kawasaki et al. 2009; Muik et al. 2009; Park et al. 2009; Yuan et al. 2009) (Fig. 1A). All of them consist of STIM1 CC2 (aa 345–391) and part of the extended CC3 domain (aa 393–437) (Muik et al. 2009), thus they are critical in the coupling to and activation of Orai1. Indeed, CAD and Orai1 have been shown to interact directly (Park et al. 2009). Furthermore, CAD has been detected to form a strong interaction with Orai1 C-terminus, while only a weaker interaction has been detected with the Orai1 N-terminus (Derler et al. 2013a; Fahrner et al. 2018a; Park et al. 2009). A potential interaction of a STIM1 C-terminal fragment with the second cytosolic segment of Orai1, the loop2, which connects TM2 and TM3, is currently controversial (Fahrner et al. 2018a; Park et al. 2009).

Structural resolution of the SOAR by NMR has uncovered key interaction sites of bound STIM1 C-terminal fragments (aa 312–387) (Stathopulos et al. 2013). Several residues have been found to participate in this STIM1/Orai1 association, including L347, L351 of one CC2 domain, and Y362, L373 and A376 of the second CC2 within a dimer of two STIM1 C-terminal fragments (Stathopulos et al. 2013) (Fig. 1B). Moreover, a positively charged cluster of residues (K382, K384, K385 and K386) seems to play an additional role in STIM1/Orai1 coupling (Stathopulos et al. 2013) (Fig. 1B). In support previously reported findings have shown that point mutations of this region (L347R, L351R, L373S, A376K) impair STIM1–Orai1 binding (Frischauf et al. 2009; Stathopulos et al. 2013).

Moreover, a recent study demonstrates that the α2 (aa 393–398) domain of the SOAR fragment, positioned between CC2 and CC3, participates in coupling to Orai1 as well as activation of store-operated Ca^2+^ entry (SOCE) (Wang et al. 2014). Those findings imply that the non-conserved amino acid F394 of STIM1 serves as a potential, direct interaction site of Orai1 (Wang et al. 2014). F394 is proposed to interact either with the Orai1 N-terminus or the hinge plate, a region that is formed by hydrophobic interactions of two leucines within TM3 (L174) and TM4 (L261) at the more cytoplasmic side of Orai1 (Wang et al. 2014; Zhou et al. 2017).

Altogether, SOAR, the C-terminal part of STIM1, serves as an essential binding site for Orai1 channels. Whereas the coupling of STIM1 C-terminal residues to the Orai1 C-terminus has been characterized in detail, those that might be involved in a potential direct or indirect interactions with other Orai cytosolic sites are still missing.

#### Essential coupling domains within Orai1

The main and indispensable coupling site for STIM1 C-terminus is the Orai1 C-terminus. Two hydrophobic residues L273, L276 in Orai1 C-terminus have been discovered to be essential (Li et al. 2007; Muik et al. 2008). Indeed, upon their point mutations to more hydrophilic amino acids (S or D) STIM1 coupling to Orai1 has been detected to be fully impaired (Li et al. 2007; Muik et al. 2008). In contrast to Orai1, Orai2 and Orai3 C-termini possess a 15–17-fold higher probability for forming a coiled–coil region (Frischauf et al. 2009). Thus, their double-point mutations are required to abolish the communication with STIM1 (Frischauf et al. 2009; Muik et al. 2008). These observations suggest distinct affinities of STIM1 to Orai1 compared to Orai2 and Orai3 and point to a probable isoform-specific Orai channel activation, which, however, requires still further proof. Additional data, based on the structure of the STIM1–Orai1 association pocket (SOAP), have revealed the importance of residues R281, L286, and R289 in STIM1 coupling (Stathopulos et al. 2013). In accordance with the dOrai crystal structure, the C-termini of two adjacent subunits are crossed in an antiparallel manner (Hou et al. 2012). Crosslinking of L273C and L276C abolishes STIM1-triggered Orai1 channel activation which suggests that the channel is likely locked in the inactive state (Tirado-Lee et al. 2015). Opening of the Orai channel is probably accompanied by a straightening of TM4-C-terminus as it has been proposed by recent crystallographic resolutions (Hou et al. 2018). Single-point mutations within the nexus region (aa 261–265) (Hou et al. 2012) have been shown to lead to constitutively active Orai1 channels (L261A, V262N H264G, K265A; Orai1 ANSGA), which is accompanied by reduced STIM1 binding (Hou et al. 2018; Zhou et al. 2016). Altogether those findings propose that an altered Orai1 C-terminus conformation impacts both the STIM1 binding, but also the active state of the channel (Zhou et al. 2016). Thus, it is assumed that the orientation of the C-termini is essential for preserved store-operated Orai1 channel activation.

Moreover, there is evidence that Orai1 N-terminus is involved in STIM1 binding; however, to a weaker extent than the C-terminus (Li et al. 2007; Muik et al. 2008; Park et al. 2009). STIM1 C- and Orai N-terminal fragments have been discovered in in vitro studies to interact directly (Derler et al. 2013a; Park et al. 2009). However, an Orai1 C-terminal deletion mutant lacks STIM1 binding (Muik et al. 2008). This suggests that if there is STIM1 binding to the Orai1 N-terminus, an indispensable requisite for that is an initial binding of STIM1 to the Orai1 C-terminus. N-terminal deletion or point mutations within the ETON region (aa 73–90) resulted in a reduction, but not fully abolished STIM1 binding. Thus, it is assumed that Orai1 N-terminus is involved in the gating process either via direct or indirect interaction with STIM1 (Derler et al. 2013a; Lis et al. 2010).

In extension to those studies, Orai1 loop2 seems to be involved in STIM1 coupling (Fahrner et al. 2018a). By employing a chimeric approach, we have discovered that non-functional Orai1 N-truncation mutants recover activation upon the exchange of Orai1 loop2 by that of Orai3 (Orai1 Δ*N*_1–78_ Orai3-L2) (Fahrner et al. 2018a). Those observations are accompanied by an enhanced STIM1 binding to the reactivated N-truncated Orai1–Orai3–loop2 chimeras (Fahrner et al. 2018a). This suggests that the loop2 of the two isoforms possesses distinct affinities to STIM1. However, the interaction studies of a STIM1 fragment with loop2 segments of Orai1 and Orai3 revealed comparable binding (Fahrner et al. 2018a). The reason for loss-of-function of Orai1 N-truncation mutants is an altered structure of the loop2 regions of Orai1 and Orai3, as revealed by MD simulation studies (Fahrner et al. 2018a). Despite an identical number of cytosolic residues connecting TM2 and TM3, the cytosolic helical extension of TM2 is shorter in Orai1 than in Orai3, resulting in a longer flexible loop2 in Orai1 compared to Orai3 (Fahrner et al. 2018a). It is likely that this more flexible region of Orai1 initiates interactions between the loop2 and the N-terminal deletion mutants. Thus, it is acting in an inhibitory manner on the channel function in an N-truncated Orai1 (Orai1 ∆*N*_1–78_), but not in Orai3 or the Orai1–Orai3–loop2 chimera (Fahrner et al. 2018a). Consequently, these distinct loop2 conformations in the Orai isoforms have been suggested to affect primarily the Orai channel conformation and only in a second step STIM1 binding, either in a direct or indirect way. If either the Orai1 N-terminus, the loop2 or both domains in a complex form a STIM1 binding site, still requires further proof. Recently reported studies on CRAC channels in *C. elegans* have shown a distinct gating mechanism compared to that in mammals (Kim et al. 2018). It is of note, that cSTIM or cCAD binding to the loop2 of cOrai has been confirmed as sufficient to gate the channel (Kim et al. 2018). This indicates that the SOCE gating process has evolved over time between different organisms.

STIM1 and Orai1 are key players that fully reconstitute CRAC channel activation (Zhou et al. 2010a), nevertheless accessory proteins further participate in or adjust their interplay. The latter includes calmodulin (CaM), CRACR2A, SPCA2, or lipids like cholesterol (Bohorquez-Hernandez et al. 2017; Derler et al. 2016b; Feng et al. 2010; Mullins et al. 2009; Pacheco et al. 2016; Park et al. 2009; Srikanth et al. 2010a).

### Potential conformational changes of the Orai channel from the closed to the open state

As already outlined in previous chapters, activation of Orai channels is initiated via STIM1 coupling to Orai1 C-terminus. Orai pore opening has been reported to be accompanied by a rotation of TM1 around the hydrophobic cavity. It has been proposed that in the active state, G98 moves into the pore, while F99 is moved out of the pore (Yamashita et al. 2017). Additionally, it has been suggested that R91 in the basic region is shifted outwards of the pore to allow Ca^2+^ influx (Frischauf et al. 2017). However, how the activation signal of STIM1–Orai1 coupling via their C-termini is transmitted to the pore region at the more N-terminal side of each Orai subunit and causes above-mentioned conformational changes in TM1 is currently unclear.

Functional and crystallographic studies have suggested that the nexus region connecting TM4 and the C-terminus extends upon STIM1–Orai1 coupling (Hou et al. 2018; Tirado-Lee et al. 2015). However, straightening of the TM4-C-terminus seems to be not sufficient for a widening of the basic region of the pore (Hou et al. 2018). The reason for that underlies the structure of an Orai1 R91S mutant, which captures a closed state and exhibits no widening of the basic pore region, but a straightened TM4-C-terminus. Thus, further conformational changes need to take place to enable pore widening within the basic region (Hou et al. 2018). At this point the question arises: how is the signal of STIM1 binding to Orai1 C-terminus propagated to the basic region of the pore? In the following paragraph, we aim to draw the potential mechanisms that Orai channel pore opening might underlie.

One hypothetical model involves STIM1 binding not only to Orai1 C-terminus but also to the cytosolic loop2 and/or the N-terminus (Fahrner et al. 2018a). A recent study by Palty and Isacoff (2016) has clearly shown by local enrichment of SOAR domains, via attaching them directly to either Orai1 N- or C-terminus, that both Orai1 N- and C-terminus participate in STIM1-triggered Orai1 activation. Their results evolved the hypothesis of stepwise STIM1–Orai1 coupling which is initiated by STIM1 binding first to Orai1 C-terminus and further to the N-terminus (Palty and Isacoff 2016). Alternatively, it might be assumed that the two cytosolic strands form a novel STIM1 binding site, which, however, requires structural resolution of a STIM1–Orai1 complex, for full proof (Palty and Isacoff 2016). An alternative hypothesis represents that STIM1 binding to Orai1 C-terminus initiates a signal propagation from the nexus region via all Orai1 TM domains, finally to the pore enabling its opening (Zhou et al. 2016). Indeed, all TM domains of Orai1 contain in total around ~ 20 residues, which are all involved in keeping Orai1 in the closed state. Their single-point mutation can lead to constitutively active channels independent of STIM1 (Derler et al. 2018; Frischauf et al. 2017; McNally et al. 2012; Palty et al. 2015; Yamashita et al. 2017; Yeung et al. 2018) (Table 1). Both hypotheses represent plausible explanations for transferring the activation signal from the C-terminus to the pore region; however, require still further proof.

### Stoichiometric requirements of the functional STIM1/Orai1 complex

The functional Orai channel complex is currently considered to possess a hexameric stoichiometry, as revealed by crystallographic resolutions of dOrai proteins as well as hOrai1 concatemeric studies (Hou et al. 2012, 2018). Thus, on the one hand one possible hypothesis represents that within one functional STIM1–Orai1 complex six STIM1 molecules are coupled to the Orai hexamer. Indeed, NMR investigations by Stathopulos et al. (2013) imply that a dimer of STIM1 C-terminal fragments binds to two Orai1 C-terminal strands. Those findings indicate a STIM1:Orai1 stoichiometry of 1:1, the so-called bimolecular interaction. On the other hand, a novel study by Zhou et al. (2017) proposed that two monomers within one STIM1 dimer bind to single Orai1 subunits of two different Orai1 channels, thus inducing a clustering of Orai channels. This assumption arose from super-resolution microscopy and FRAP studies on cells expressing Orai1 together with a dimer of two STIM1 C-terminal fragments (Zhou et al. 2017). Only wild-type dimers exhibited clustering of Orai1 channels, while a dimer with one mutated subunit exhibited binding and current activation, but no clustering. Thus, they assume that functional STIM1–Orai1 coupling is accompanied by clustering of Orai channels via the two monomers of a STIM1 dimer. This study indicates a global 1:1 stoichiometry of STIM1–Orai proteins, thus termed unimolecular binding. An alternative hypothesis suggests that a 2:1 STIM1:Orai1 ratio leads to the maximal Orai1 activation (Hoover and Lewis 2011; Li et al. 2011; Palty et al. 2017; Scrimgeour et al. 2009, 2014). This indicates that twelve STIM1 molecules would be necessary for full Orai hexamer activation. Palty et al. (Palty et al. 2017) proposed that this 2:1 ratio is probably manifested by a sequential Orai1 activation. First, a STIM1 dimer binds to an Orai1 C-terminus resulting in conformational changes within the Orai1 channel leading to a partially opened channel complex. In the next step, the second C-terminus of the STIM1 dimer couples to the same Orai1 subunit, finally enabling full Orai1 activation (Palty et al. 2017). Nevertheless, at this stage it remains elusive if the second STIM1 monomer within the dimer structure additionally couples to either the C-terminus or another cytosolic site of the Orai1 channel. Altogether, the stoichiometry of an active CRAC channel complex remains controversial; however, the amount of bound STIM1 molecules has been discovered to play an essential role in maintaining typical CRAC channel biophysical characteristics (Scrimgeour et al. 2009, 2014) as outlined in detail in “Role of STIM1 in maintaining authentic CRAC channel hallmarks”

### Biophysical Characteristics of CRAC Channels

**Fig. 2:**
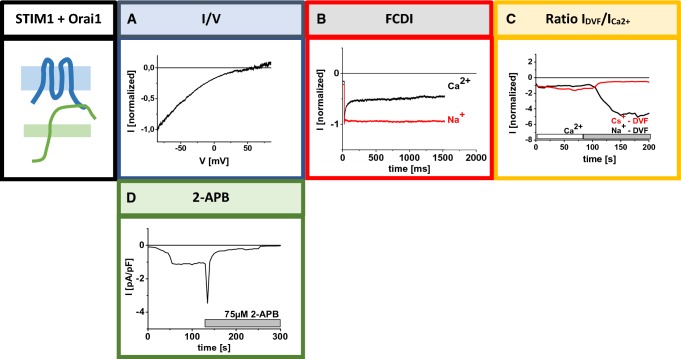
Authentic CRAC channel hallmarks of STIM1/Orai1 currents. (**A**) Current/voltage (*I*/*V*) relationship with a reversal potential (*V*_rev_) > 0. (**B**) FCDI in a Ca^2+^-containing, Ba^2+^-containing compared to a Na^+^-containing divalent-free solution. (**C**) Alterations of currents upon the switch from a Ca^2+^ containing to a Na^+^- or Cs^+^-containing divalent-free solution (ratio of *I*_DVF_/*I*_Ca2+_). (**D**) Effects of 75 µM 2-APB upon full activation of STIM1/Orai1 currents Graphs adapted from (Derler et al. 2018)

Unique biophysical characteristics of CRAC channels are, very high Ca^2+^ ion selectivity, extremely small unitary conductance, fast Ca^2+^-dependent inactivation, enhancements in *I*_DVF_ (currents in a Na^+^-containing divalent-free solution) compared to *I*_Ca2+_ (currents in a Ca^2+^ containing solution) and modulation by 2-APB (Fig. 2) (McNally et al. 2009; Prakriya and Lewis 2006), which will be described in more detail in the following.

#### Ca^2+^ selectivity

Among the broad family of Ca^2+^ ion channels (e.g. L-type, TRPV6, ryanodine receptors), CRAC channels are most selective for Ca^2+^. The CRAC channel currents appear in strong inward rectification exhibiting a reversal potential of ~ + 50 mV (Hoth and Penner 1992; Lis et al. 2007) (Fig. 2A), which can be determined of an *I*/*V* trace obtained upon application of a voltage ramp at maximal CRAC current activation (Supplementary Figure 1A). This high Ca^2+^ selectivity likely arises due to ion–pore and ion–ion interactions (Prakriya 2009). They exhibit a 1000 times-enhanced permeation for Ca^2+^ than for the monovalent ion Na^+^ (Bakowski and Parekh 2002; Hoth and Penner 1993; Lepple-Wienhues and Cahalan 1996; McCleskey and Almers 1985; Mullins et al. 2016; Prakriya and Lewis 2002; Su et al. 2004). In a divalent-free solution, monovalent ions such as Na^+^, Li^+^ and K^+^ can permeate through the channel, however, in the presence of micromolar Ca^2+^ concentrations Orai channel currents are blocked (Bakowski and Parekh 2002; Lepple-Wienhues and Cahalan 1996; McCleskey and Almers 1985; Mullins et al. 2016; Prakriya and Lewis 2002; Su et al. 2004). Unlike other Ca^2+^ ion channels, CRAC channels are unable to conduct Cs^+^ ions due to the very narrow pore diameter in the range of 3.8–3.9 Å (McCleskey and Almers 1985; Yamashita et al. 2007). Further, CRAC channels exhibit a 20-fold lower binding affinity for Ca^2+^ (Sather and McCleskey 2003). It is, however, compensated by a slow rate of ion flux which justifies their high Ca^2+^ selectivity (Yamashita et al. 2007; Yamashita and Prakriya 2014). Furthermore, high Ca^2+^ selectivity of CRAC channels likely arises due to the very narrow pore diameter, as proposed by simulations studies, that find an inverse correlation of Ca^2+^ selectivity versus pore volume (Boda et al. 2007; Malasics et al. 2009). Structurally, high Ca^2+^ selectivity of Orai channels is defined by TM1, TM3 and extracellular loops of the Orai channel. The residues E106 within all six TM1s assemble to a ring of glutamates and form the selectivity filter at the exterior of the pore mouth. The function of the selectivity filter is supported by the three negatively charged residues in CAR (Frischauf et al. 2015). Electrostatic interactions between the CAR in TM1–TM2 loop1 and the TM3–TM4 loop3 have been detected to fine-tune the Ca^2+^ accumulation towards the pore and, in consequence, ion permeation (Frischauf et al. 2015). Additionally, the residue E190 in TM3 controls high Ca^2+^ selectivity of the Orai1 channel as its single-point mutation to a glutamine or alanine (E190Q/A) leads to an increased pore diameter of around 7 Å and thus, an increased permeation of Cs^+^ ions (Zhou et al. 2010b). New MD studies have proposed that dOrai W262Q (analogous position to hOrai1 E190Q) effects the hydration pattern of the pore and the dynamics of the surrounding residues in TM3 (Alavizargar et al. 2018).

#### Unitary conductance

Another distinctive characteristic of CRAC channels is their extremely small unitary conductance in the range of 9–24 fS in a 2–110 mM Ca^2+^ solution (Prakriya and Lewis 2002; Zweifach and Lewis 1993). So far, due to this attribute no single-channel CRAC current recordings were feasible (Prakriya and Lewis 2003, 2006). The reason for such small unitary conductance probably underlies the very narrow pore diameter in contrast to other Ca^2+^ ion channels (Boda et al. 2007; Malasics et al. 2009). Nevertheless, several studies approximated the association rate of Ca^2+^ for the CRAC channel pore-blocking site around 4 × 10^6^ M^−1^ s^−1^ (Prakriya and Lewis 2006; Yamashita et al. 2007). This feature suggests that Ca^2+^ entry from the extracellular space towards the selectivity filter of the channel is strictly limited, which could potentially clarify the small conductance of the channel. The small conductance of the CRAC channel is of interest for further investigations as it enables relatively specific activation of Ca^2+^-dependent downstream signalling pathways (Di Capite et al. 2009).

#### Fast Ca^2+^-dependent inactivation

The next outstanding CRAC channel characteristic is Ca^2+^-dependent inactivation (CDI) which controls the inhibition of Orai/CRAC channel activity. The CDI serves as an essential feedback regulation mechanism of cytosolic Ca^2+^ levels. There are two types of Ca^2+^-dependent inactivation: the fast (FCDI) (Fig. 2B) and the slow (SCDI) one (Zweifach and Lewis 1995a, b). The FCDI is triggered by Ca^2+^ that permeates through the channel and binds to a specific locus within a short distance from the cytosolic pore mouth eliciting a rapid inactivation of the channel over tens of milliseconds (Hoth and Penner 1993; Zweifach and Lewis 1995a). It is monitored as a decrease in CRAC currents during a hyperpolarizing voltage step applied over tens of milliseconds (Hoth and Penner 1993; Prakriya and Lewis 2003; Zweifach and Lewis 1995a) (Supplementary Figure 1D). In extension, the slow inactivation occurs in the range of seconds and can last for several minutes for full completion (Zweifach and Lewis 1995b). SCDI is monitored upon application of repetitive voltage ramps and represents a phase subsequent to maximal activation of CRAC currents (Supplementary Figure 1C). CRAC channels’ CDI is regulated not only by STIM1 and Orai1 but also additional accessory proteins, CaM and SARAF (Lopez et al. 2016).

#### Enhancements in *I*_DVF_ compared to *I*_Ca2+_

Another prominent CRAC channel characteristic is the increase in the current levels in *I*_DVF_ compared to *I*_Ca2+_ (Fig. 2C) as monitored upon application of repetitive voltage ramps (Supplementary Figure 1B). It has been assumed that this unique current enhancement corresponds to the intensity of CDI (Prakriya and Lewis 2006). CRAC channels exhibit FCDI in a Ca^2+^-containing solution; however, in a divalent-free Na^+^-containing solution inactivation is lost (Prakriya and Lewis 2006). This potentially explains the increase in current densities in a Na^+^- versus Ca^2+^-containing solution as a charge carrier (Prakriya et al. 2006). Several studies have indicated that FCDI likely lowers the opening probability when Ca^2+^ rather than Na^+^ ions are the charge carriers (Mullins and Lewis 2016; Mullins et al. 2016; Prakriya and Lewis 2006; Zweifach and Lewis 1995a). Thus, an increase in *I*_DVF_ versus *I*_Ca2+_ is potentially governed by a decreased open probability in a Ca^2+^-compared to a Na^+^-containing solution (Prakriya and Lewis 2006).

#### Modulation by 2-APB

Additionally, CRAC channels possess a unique behaviour upon application of the pharmacological modulator 2-aminoethyldiphenyl borate (2-APB) (Fig. 2D). Surprisingly, at low concentrations (1–5 µM) 2-APB induces a persistent two–fivefold increase in *I*_CRAC_, whereas at higher concentration (> 10 µM) a transient increase of *I*_CRAC_ occurs but is followed by immediate strong inhibition (Derler et al. 2008; Prakriya and Lewis 2001). It is of note, that electrophysiological experiments of *I*_CRAC_ revealed that the high concentrations of 2-APB induce loss of FCDI (Prakriya and Lewis 2001). The latter might result from conformational changes within the CRAC channel. Although the effects of 2-APB are quite well defined for CRAC channels, the mode of action for this modulator is still not understood.

### Key determinants involved to maintain authentic CRAC channel hallmarks

In the following we provide an overview on the diversity of factors which determine the maintenance of store-operated activation and of authentic CRAC channel hallmarks, with special focus on *V*_rev_, FCDI, the ratio *I*_DVF_:*I*_Ca2+_ and partly the effects of 2-APB.

#### Role of STIM1 in maintaining authentic CRAC channel hallmarks

**Fig. 3:**
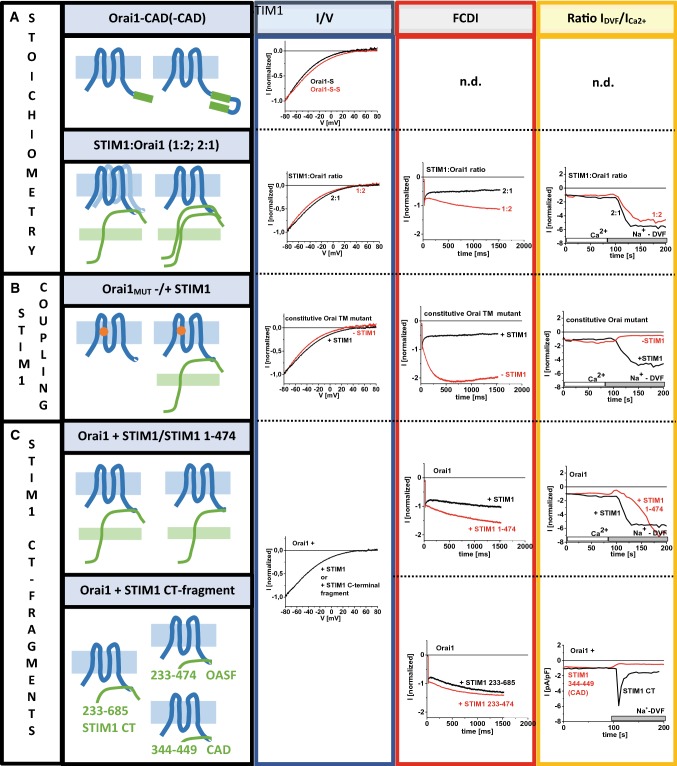
Role of STIM1 in the maintenance of the CRAC channel hallmarks. (**A**) Effect of the stoichiometry of STIM1:Orai1 expression, (**B**) of the coupling of STIM1 to Orai1 and (**C**) of certain STIM1 C-terminal fragments on the *V*_rev_, FCDI and the ratio of *I*_DVF_:*I*_Ca2+_. Regarding the stoichiometry Orai1 attached to one or two CAD fragments (Prakriya et al. 2006) and the expression of STIM1:Orai1 with a ratio of 2:1 and 1:2 (Scrimgeour et al. 2009, 2014) are compared (**A**). Constitutively active Orai1 mutants are used to show that functional coupling of STIM1 is required for the maintenance of CRAC channel hallmarks (**B**). Regarding STIM1 fragments, a STIM1 C-terminal deletion mutant and some STIM1 C-terminal fragments are compared to wild-type STIM1 mediated Orai1 currents in respect to the maintenance of the CRAC channel hallmarks. Graphs are adapted from (Prakriya et al. 2006; Scrimgeour et al. 2009) or Supplementary Figure 2

STIM1 contributes to the maintenance of authentic CRAC channel hallmarks through (i) its binding to Orai channels (McNally et al. 2012), (ii) its stoichiometry (Scrimgeour et al. 2009; Scrimgeour et al. 2014) together with Orai proteins in a functional STIM1/Orai1 complex as well as (iii) specific segments in its C-terminus (Derler et al. 2009b; Mullins et al. 2009; Yuan et al. 2009) (Fig. 3A–C).

Ad (i) high Ca^2+^-selective Orai1 Ca^2+^ currents with a reversal potential in the range of + 50 mV (Hoth and Penner 1993; Yeromin et al. 2006) are ensured by functional coupling of STIM1 (Derler et al. 2018; McNally et al. 2012; Palty et al. 2015; Yeung et al. 2018) (Tables 1, 2). This has been shown by a diversity of constitutively active Orai TM point mutants (such as Orai1 V102A, Orai1 P245L) (Derler et al. 2018; McNally et al. 2012; Palty et al. 2015; Yeung et al. 2018) (Tables 1, 2). They possess poor Ca^2+^ selectivity in the absence of STIM1, which can be fully restored only in the presence of STIM1 and upon store-depletion (Derler et al. 2018; McNally et al. 2012; Palty et al. 2015; Yeung et al. 2018). The reversal potential of these constitutive mutants is only in the presence of STIM1 in the range of + 50 mV, while in the absence of STIM1 it is shifted to more negative potentials in the range of + 10 to + 30 mV (Fig. 3B) (Table 2).

**Table 2:** List of Orai mutants differentiated by their biophysical hallmarks

Mutation	*V*_rev_ [mV]		Inactivation		*I*_DVF_:*I*_Ca2+_		References
	+ STIM	− STIM	+ STIM	− STIM	+ STIM	− STIM	
*Orai 1*							
Wildtype	52.3 ± 2.8	–	FCDI, followed by reactivation	–	> 1	–	Derler et al. (2018), Yeung et al. (2018)
S97C	50.3 ± 0.9	37.3 ± 4.4	n.d.	Reactivation	n.d.	n.d.	Yeung et al. (2018)
V102A	61.7 ± 6.0	21.5 ± 3.3	FCDI	Reactivation	< 1	< 1	McNally et al. (2012)
V102C	74.6 ± 2.3	18.3 ± 1.4	FCDI	Reactivation	< 1	< 1	McNally et al. (2012)
H134A	59.9 ± 3.3	49.1 ± 4.2	n.d.	n.d.	> 1	> 1	Yeung et al. (2018)
H134C	57.2 ± 3.7	42.0 ± 1.9	n.d.	Reactivation	n.d.	n.d.	Yeung et al. (2018)
L185A F250A	55 ± 4	35 ± 3	FCDI, followed by reactivation	Reactivation	> 1	< 1	Derler et al. (2018)
F187C	54.1 ± 2.3	39.0 ± 3.2	n.d.	Reactivation	n.d.	n.d.	Yeung et al. (2018)
A235C	58.7 ± 1.2	51.4 ± 4.2	n.d.	Reactivation	n.d.	< 1	Yeung et al. (2018)
P245L	69.5 ± 4.4	37.7 ± 6.7	FCDI	Reactivation	< 1	> 1	Derler et al. (2018), Palty et al. (2015)
Δ1–71 O3–L2	n.d.	–	FCDI, followed by reactivation	–	< 1	–	Derler et al. (2018)
Δ1–72 O3–L2	n.d.	–	FCDI, followed by reactivation	–	< 1	–	Derler et al. (2018)
Δ1–74	48 ± 5	–	No FCDI	–	< 1	–	Derler et al. (2018)
Δ1–78 O3–L2 P245L	32 ± 4	35 ± 3	Reactivation	Reactivation	> 1	> 1	Derler et al. (2018)
*Orai 3*							
Wildtype	70 ± 9	–	FCDI	–	> 1	–	Derler et al. (2018)
F160A	52 ± 7	48 ± 4	FCDI	Reactivation	< 1	> 1	Derler et al. (2018)
P254L	55 ± 5	43 ± 3	FCDI	Reactivation	< 1	> 1	Derler et al. (2018)
Δ1–46	49 ± 9	–	FCDI	–	< 1	n.d.	Bergsmann et al. (2011), Derler et al. (2018)
Δ1–49	45 ± 5	–	Reactivation	–	> 1	n.d.	Bergsmann et al. (2011), Derler et al. (2018)
Δ1–51	17 ± 3	–	FCDI, followed by reactivation	–	n.d.	n.d.	Bergsmann et al. (2011)
Δ1–53 F160A	35 ± 4	25 ± 1	Reactivation	Reactivation	> 1	> 1	Derler et al. (2018)
Δ1–46 F160A	57 ± 3	–	FCDI	n.d.	< 1	n.d.	Derler et al. (2018)
Δ1–53 P254L	25 ± 3	32 ± 3	Reactivation	Reactivation	> 1	> 1	Derler et al. (2018)

Fast Ca^2+^-dependent inactivation is another dynamic property of CRAC channels as it has been also shown by constitutively active Orai TM mutants. Only in the presence of STIM1, FCDI is fully maintained comparable to wild-type Orai1 channels. In the absence of STIM1, these mutants exhibit, instead of FCDI, strong reactivation. This phenomenon probably occurs due to a less-selective pore of Orai1 mutants. Only in the presence of STIM1, constitutive Orai mutants display enhanced selectivity and in-line FCDI occurs instead of reactivation. This suggests that STIM1 binding fine-tunes and stabilizes the pore architecture of several constitutively active Orai mutants (Derler et al. 2018; McNally et al. 2009; Palty et al. 2015; Yamashita et al. 2007; Yeung et al. 2018) (Fig. 3B) (Table 2).

Moreover, also the enhancement of *I*_DVF_ versus *I*_Ca2+_ is governed by STIM1 (Derler et al. 2013a, 2018; McNally et al. 2013). Non-selective, constitutively active mutants exhibit only in the presence of STIM1 a ratio *I*_DVF_:*I*_Ca2+_ > 1 (Fig. 3B) (Table 2). Based on the previous hypothesis (Prakriya and Lewis 2006), this prominent increase in currents can possibly be explained by strong FCDI in a Ca^2+^-containing solution and loss of FCDI in a divalent-free Na^+^-containing solution of these constitutively active mutants in the presence of STIM1 (Derler et al. 2018; Prakriya et al. 2006). As suggested for native CRAC currents, this correlation underlies probably an altered open probability in a Ca^2+^- versus Na^+^- containing solution (Prakriya and Lewis 2006). However, further studies are still required to support these assumptions. In the absence of STIM1, *I*_DVF_ of constitutively active mutants is either equal or smaller compared to *I*_Ca2+_ (Derler et al. 2018), which will be highlighted in more detail in the following chapter.

Constitutive mutants containing the C-terminal single point mutation L273D/S, L276D/S, which abolishes coupling to STIM1, exhibit a loss of typical CRAC channel properties (Derler et al. 2013a, 2018; McNally et al. 2013). Thus, functional coupling of STIM1 to Orai channels is an indispensable requisite to maintain authentic CRAC channel hallmarks.

Ad (ii) furthermore, typical CRAC channel hallmarks are determined by the ratio of STIM1:Orai1 proteins when associated as complex (McNally et al. 2012; Scrimgeour et al. 2009, 2014) (Fig. 3A). This has been proven on the one hand by Orai1 subunits tethered to STIM1 C-terminal fragments. Ion channels composed of such constructs exhibit enhancing Ca^2+^ ion selectivity of wild-type Orai1 currents with increasing amount of bound STIM1 (McNally et al. 2012) (Fig. 3A). On the other hand, STIM1/Orai1 currents of cells expressing distinct ratios of STIM1 and Orai1 have been shown to lead to alterations in CRAC channel hallmarks. FCDI is much stronger pronounced for a high STIM1:Orai1 ratio compared to a low ratio (Scrimgeour et al. 2009). High levels of STIM1 versus Orai1 exhibit currents with a FCDI composed of a fast inactivation phase occurring within the first 200 ms after application of a hyperpolarizing voltage-step and a subsequent slow inactivation phase (Fig. 3A). Lower levels of STIM1 versus Orai1 exhibit FCDI within the first 50–100 ms which is followed by a reactivation phase (Scrimgeour et al. 2009, 2014). The reversal potential has been reported to vary in dependence on the extent of FCDI (Scrimgeour et al. 2009). Further, currents of both high and low ratio of STIM1:Orai1 expression in a Ca^2+^-containing solution are enhanced upon the switch to a Na^+^-containing divalent-free solution (Fig. 3A). Nevertheless, Na^+^ currents under high STIM1:Orai1 ratio conditions have been shown to activate faster than those of cells expressing a lower ratio (Scrimgeour et al. 2009). Additionally, dependence of the extent of the *I*_DVF_:*I*_Ca2+_ on the STIM1:Orai1 expression ratio has been observed for the constitutively active Orai1 P245L mutant. The more STIM1 is expressed, the higher and faster is the enhancement in *I*_DVF_ compared to *I*_Ca2+_ when co-expressed with STIM1 in Orai1 KO cells (Supplementary Figure 2A). Moreover, differential effects have been observed for the CRAC channel inhibitor 2-APB. The transient activation of STIM1-mediated Ca^2+^ currents occurs at a lower level for cells containing a high STIM1:Orai1 ratio compared to those with a lower one (Scrimgeour et al. 2009).

Ad (iii) additionally, maintenance of CRAC channel hallmarks is determined by segments within STIM1 C-terminus. Small STIM1 C-terminal fragments, such as CAD or SOAR are sufficient for Orai channel activation independent of store-depletion in a constitutive manner (Derler et al. 2009b; Kawasaki et al. 2009; Park et al. 2009; Yuan et al. 2009), but not for maintenance of CRAC channel hallmarks. Reversal potentials of constitutively active currents mediated by STIM1 fragments are comparable to those of wild-type STIM1/Orai1 currents; however, FCDI and enhancement in *I*_DVF_ versus *I*_Ca2+_ differ (Derler et al. 2009b) (Fig. 3C). While FCDI of Orai1 currents activated by STIM1 C-terminus (233–685) exhibit comparable extents as that of full-length STIM1, currents stimulated by the minimal activating fragments [OASF (aa 233–474), CAD (aa 344–449)] lose FCDI (Derler et al. 2009b). In line, an enhancement in *I*_DVF_ versus *I*_Ca2+_ has been observed for Orai1 currents activated by the entire STIM1 C-terminus (Fig. 2C, Supplementary Figure 2C), but not for OASF or CAD (Fig. 2C, Supplementary Figure 2D, E). Orai1 Ca^2+^ currents activated by a STIM1 C-terminal deletion mutant, STIM1 1–474 also led to constitutive activity with loss of FCDI (Derler et al. 2009b). Intriguingly, the ratio of *I*_DVF_:*I*_Ca2+_ is higher than 1 for STIM1 1–474/Orai1 currents, but still developed slower than for wild-type STIM1–Orai1 currents (Fig. 3C, Supplementary Figure 2B). Thus, the lack of a negatively charged cluster (aa 474–485), termed CRAC modulatory domain (CMD), has been attributed to a loss of FCDI (Derler et al. 2009a). In contrast, maintenance of an enhancement of *I*_DVF_ versus *I*_Ca2+_ seems to be not solely triggered by the CMD, but to require also structural portions of STIM1 N-terminus or the TM domain.

Sole constitutive activity of STIM1 mediated Orai currents does not lead to an altered FCDI and ratio of *I*_DVF_:*I*_Ca2+_, as it has been shown by STIM1 EF-hand mutants, STIM1 D76A/D76A E87A (Derler et al. 2009b). Ca^2+^ binding leads to constitutive association to and activation of Orai channels, however, with still maintained FDCI and a ratio of *I*_DVF_:*I*_Ca2+_ > 1 (Spassova et al. 2008). It remains to be determined if a STIM1 D76A lacking CMD, alters FCDI together with the ratio of *I*_DVF_:*I*_Ca2+_. Recently discovered mutants STIM1 R304W (Fahrner et al. 2018b) and STIM1 L251S (Fahrner et al. 2014), respectively, have been found to exhibit constitutive activity. Hence, also these mutants require further testing in regard of the maintenance of CRAC channel hallmarks.

In summary, the maintenance of the typical CRAC channel hallmarks is only guaranteed upon functional coupling of STIM1 to Orai1, higher expression levels of STIM1 compared to Orai1 and STIM1 CMD. Furthermore, other STIM1 domains might be involved in the control of typical CRAC channel properties, which, however, requires still continuing investigations. As not all STIM1 mutants, which exhibit loss in FCDI, show also alterations in the ratio of *I*_DVF_:*I*_Ca2+_, it is likely that the individual CRAC channel properties depend on the distinct structural features of STIM1.

#### Role of Orai1 in maintaining CRAC channel hallmarks

**Fig. 4:**
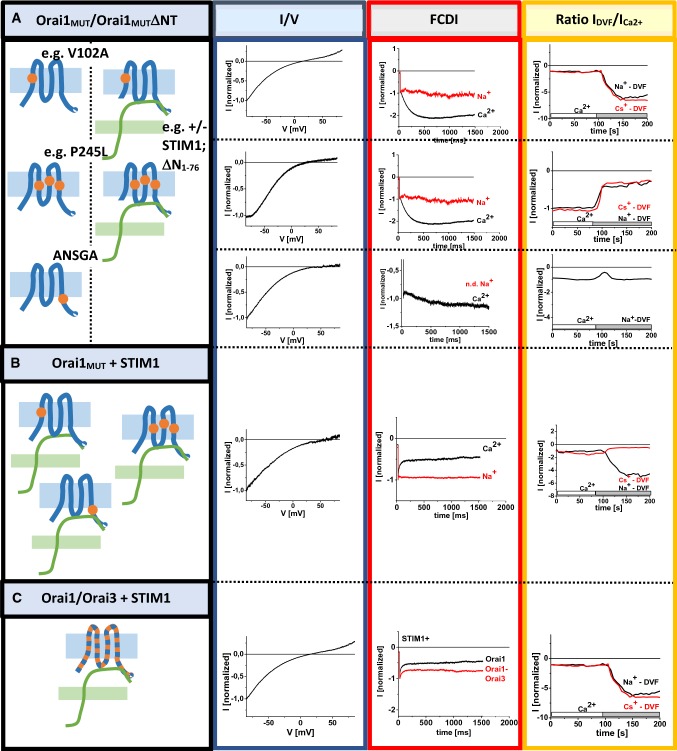
Role of Orai1 in the maintenance of the CRAC channel hallmarks. (**A, B**) Effects of Orai1 TM and N-terminal mutants in the absence or presence of STIM1 and (C) of Orai heteromerization on the *V*_rev_, FCDI and the ratio of *I*_DVF_:*I*_Ca2+_. Orai1 TM mutants include, for example Orai1 V102A, Orai1 F136S, Orai1 P245L or Orai1 ANSGA (Palty et al. 2015; Prakriya et al. 2006). Orai1 N-terminal deletion mutants that lose typical CRAC channel hallmarks represent Orai1 Δ*N*_1–74_ or shorter ones. Only in the presence of STIM1 and an intact Orai1 N-terminus (at least the first 72 residues) authentic CRAC channel hallmarks are maintained (**A, B**) (Derler et al. 2018). In respect to Orai protein heteromerization Orai1 and Orai3 isoforms are shown (**C**) (Schindl et al. 2009). Graphs adapted from Derler et al. (2018) and Schindl et al. (2009)

CRAC channel hallmarks are further controlled by (i) segments as well as single residues within the Orai channel proteins and (ii) whether Orai subunits form homo- or heteromeric channels (Fig. 4A–C).

Ad (i) among segments within the Orai channel, the N-terminal proline-/arginine-rich region has been reported to control the phases of inactivation upon application of a hyperpolarizing voltage step. STIM1 mediated Orai1 currents typically show a fast inactivation phase followed by reactivation. The latter is abolished upon truncation of this proline-/arginine-rich region (Frischauf et al. 2011).

**Fig. 5:**
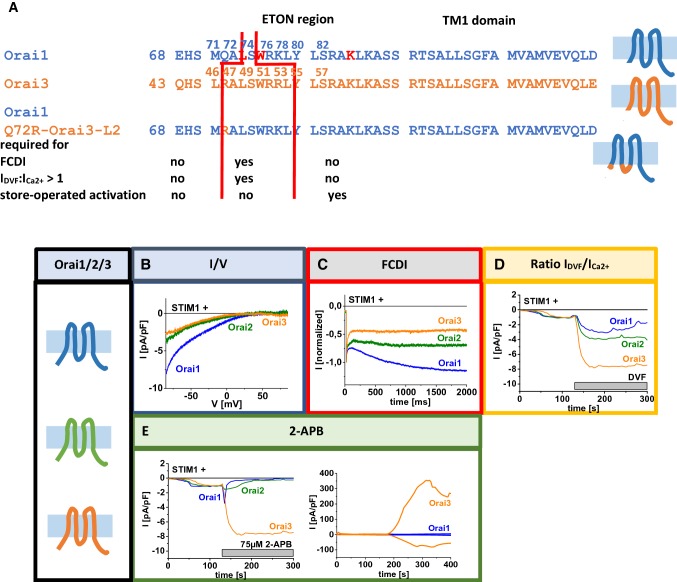
Isoform-specific differences in the CRAC channel hallmarks. (**A**) Sequence alignement of Orai1, Orai3 and Orai1 Q72R Orai3–L2 of the ETON and TM1 region. Red lines mark the parts of the ETON region required for maintenance of store-operated activation, FCDI and a ratio of *I*_DVF_:*I*_Ca2+_ > 1. (**B–D**) Comparison of the authentic CRAC channel hallmarks, *V*_rev_, FCDI, ratio of *I*_DVF_:*I*_Ca2+_ and effects of 2-APB on Orai1, Orai2 and Orai3. Graphs adapted from Derler et al. (2009b, 2013b), Fahrner et al. (2018a), Frischauf et al. (2009), and Lis et al. (2007)

The ETON region is another critical determinant that maintains CRAC channel characteristics (Derler et al. 2013a). A combined approach of deletion and single point mutations has revealed that distinct parts of the ETON region control either store-operated activation or the maintenance of other typical CRAC channel hallmarks (Bergsmann et al. 2011; Derler et al. 2018) (Fig. 5A). This regulation occurs in an isoform-specific manner due to a dependence of the cytosolic loop2 (Fahrner et al. 2018a) as outlined in detail in the next chapter (Fig. 5). While store-operated activation of Orai channels requires only a part of the ETON region, the whole helical N-terminal segment is essential to adjust FCDI and *I*_DVF_ > *I*_Ca2+_ (Derler et al. 2018; Fahrner et al. 2018a). This holds for wild-type Orai channels as well as constitutively active Orai mutants (Derler et al. 2018). Deletion of approximately the first half (aa 72–80 in Orai1/Orai1–Orai3–L2, aa 46–55 in Orai3) of the ETON region leads to an abolished FCDI and a decrease in *I*_Ca2+_ compared to *I*_DVF_ (Bergsmann et al. 2011; Derler et al. 2018) (Fig. 5A, Table 2). One exception represents Orai1 Δ*N*_1–74_ which still exhibits preserved FCDI, but a reduction of *I*_DVF_ compared to *I*_Ca2+_ (Derler et al. 2018). Single point mutations (L74E W76E, K85E) even uncovered that only the first half of the ETON region controls these CRAC channel hallmarks, while the second part of the ETON region is exclusively required to maintain store-operated activation (Derler et al. 2018) (Fig. 5A). Additionally, the maintenance of typical CRAC channel hallmarks occurs in an isoform-specific manner which will be highlighted in detail in the next chapter.

Moreover, the intracellular loop connecting TM2 and TM3 of Orai channels adjusts fast inactivation and reactivation, which has been demonstrated by mutational and chimeric studies (Srikanth et al. 2010b).

As determined via constitutively active Orai TM mutants, several positions within the TM domains contribute to the adjustment of CRAC channel hallmarks, especially together with STIM1. Despite the point mutations within the Orai TM domains can lead to constitutively active Orai channels, they exhibit diminished Ca^2+^ selectivity, instead of FCDI only reactivation and a decrease in *I*_DVF_ compared to *I*_Ca2+_. Only STIM1 together with an intact N-terminus is able to overcome the effects of the single point mutations, likely due to a conformational rearrangement of the Orai channel. Interestingly, constitutively active single point mutants can be roughly categorized into three groups in dependence on their effects on the CRAC channel hallmarks in the absence of STIM1: (1) Orai TM1 mutants (e.g. Orai1 V102A), (2) Orai mutants containing substitutions in either TM2, TM3 or TM4 (e.g. Orai1 F136S, Orai1 P245L) and (3) Orai hinge mutants (Orai1 ANSGA) (Derler et al. 2018) (Fig. 4A) (Butorac et al. 2018).

Orai1 V102A/C represents an Orai1 TM1 mutant. It is the least selective among all known constitutive Orai TM mutants, with a reversal potential in the range of + 20 mV in the absence of STIM1. In contrast to Orai1 V102A/C, the other constitutive Orai1 TM mutants (Orai1 F136S, Orai1 L185A F250A, Orai1 P245L) exhibit a reversal potential of + 30 to + 40 mV in the absence of STIM1 (Fig. 4A). In the presence of STIM1, all mutants reach comparable levels of *V*_rev_ as CRAC and STIM1/Orai1 mediated currents (Butorac et al. 2018; Derler et al. 2013a; McNally et al. 2012, 2013) (Fig. 4B). One exception represents the Orai1 H134A, which shows already in the absence of STIM1 a very high *V*_rev_ in the range of + 50 mV, which does not further enhance in the presence of STIM1. Also, the Orai1 nexus mutant, Orai1 ANSGA, yields a *V*_rev_ of + 50 mV already in the absence of STIM1. Hence, the latter two mutants seem to capture an open state that matches that of the STIM1-bound Orai.

Except for Orai1 ANSGA, all other constitutive Orai1 TM mutants exhibit loss of FCDI in the absence of STIM1. Instead, all mutants exhibit strong reactivation, which can be only converted to FCDI upon STIM1 binding. FCDI of Orai1 ANSGA is clearly present, however, still reduced compared to the presence of STIM1 (Fig. 4A, B).

Additionally, an enhancement in *I*_DVF_ versus *I*_Ca2+_ is lost for most of the constitutive Orai1 TM mutants (Fig. 4A), a behaviour that has been demonstrated for the first time by Derler et al. (2018). The reason for that is probably the distinct extent of inactivation found in Ca^2+^- versus Na^+^-containing solutions. In the presence of Ca^2+^, these constitutive mutants exhibit upon application of hyperpolarizing voltage steps not only loss of inactivation, but show strong reactivation. In contrast, in a Na^+^-containing divalent-free solution those mutants yield only loss of inactivation, and lack reactivation. Hence, this distinct “inactivation behavior” in a Ca^2+^-versus Na^+^-containing solution might be responsible for *I*_DVF_ < *I*_Ca2+_. While in general, constitutive Orai TM2, TM3 and TM4 point mutants exhibit a ratio *I*_DVF_:*I*_Ca2+_ < 1, the Orai1 ANSGA mutant exhibits a ratio *I*_DVF_:*I*_Ca2+_ = 1 (Fig. 4A). Only Orai1 V102A and Orai1 H134A show an enhancement of *I*_DVF_ compared to *I*_Ca2+_ (Fig. 3A) (Butorac et al. 2018). The reason for these distinct ratios is probably an altered pore diameter. CRAC or STIM1/Orai1 channels and constitutive Orai1 TM mutants, such as Orai1 P245L, are impermeable for Cs^+^ independent of the absence or presence of STIM1. In contrast, Orai1 V102A/C show Cs^+^ permeation in the absence of STIM1, but not in its presence. This indicates that the pore radius is wider in the STIM1 unbound state, but further investigations are still required to proof this assumption.

However, this distinct behaviour opens another question. Why do Orai1 V102A/C and other Orai1 TM mutants show an identical inactivation behaviour, although *V*_rev_ and *I*_DVF_/*I*_Ca2+_ are distinct (Derler et al. 2018; McNally and Prakriya 2012)? One explanation represents that an increased pore diameter, like for Orai1 V102A, does not further enhance the extent of reactivation. To clarify this point, additional evaluations are necessary.

Ad (ii) additionally, CRAC channel hallmarks are altered when Orai channels form heteromeric assemblies as it has been demonstrated for Orai1–Orai3 co-expressing cells. Heteromeric Orai1/Orai3 channels have been shown to exhibit reduced Ca^2+^ selectivity, clear Cs^+^ permeation and diminished fast inactivation (Schindl et al. 2009) (Fig. 4C). Similar to homomeric Orai channels that show an enhanced *I*_DVF_ compared to *I*_Ca2+_, currents of Orai1 and Orai3 expressing cell are also enhanced upon the switch from a Ca^2+^ containing to Na^+^-containing divalent-free solution (Fig. 4C). The reason for altered CRAC channel hallmarks in heteromeric Orai channels represents a distinct composition of the CAR region within the Orai isoforms (Schindl et al. 2009). While Orai1 contains three glutamates there, Orai2 and Orai3 exhibit a mixture of glutamates, glutamines and aspartates. Hence, in contrast to homomeric Orai channels, heteromeric forms contain an asymmetric composition of the glutamates and aspartates in the loop1 region. This can lead to less Ca^2+^ selective currents with an enhanced Cs^+^ permeation and reduced fast inactivation. Thus, the acidic Ca^2+^ coordination site in the first loop2 is not only involved in the attraction of Ca^2+^ ions, but also controls Ca^2+^ selectivity and inactivation.

Additionally, FCDI is adjusted by two pore-lining residues within the basic region of the pore of Orai1 channels (Yamashita et al. 2007). Their mutation to negatively charged residues (W76E, Y80E) completely abolished FCDI.

In summary, maintenance of typical CRAC channel properties depends on cytosolic Orai regions, pore-lining residues, amino acids within the TM domains and the composition of the Orai channel, either as a homo- or heteromer. Some positions have been discovered to be indispensable for maintenance of all hallmarks: Ca^2+^ selectivity, FCDI and the ratio of *I*_DVF_:*I*_Ca2+_. However, some regions (e.g. W74 or aa 1-74; V102) seem to control only one of those properties, while the others are retained. This underlies probably an altered structure of the Orai channel or a distinct communication of STIM1 with Orai1.

#### Orai channels exhibit isoform-specific CRAC channel hallmarks

The three Orai isoforms are all activated by STIM1 in a store-operated manner and exhibit highly Ca^2+^ selective currents with comparable reversal potentials (Lis et al. 2007). Nevertheless, they differ in diverse other biophysical characteristics due to the structural differences.

First of all, the level of Ca^2+^ entry/currents across the three isoforms is distinct. STIM1 mediated Orai1 currents are approximately two- to three-fold larger than those of Orai2 and Orai3 (Frischauf et al. 2009; Lis et al. 2007) (Fig. 5B), while *V*_rev_ is comparable in the range of + 50 mV. The reason for that has been reported to underlie a polybasic and proline–rich region at the beginning of the N-terminus only occurring in Orai1, but not in Orai2 and Orai3 (Fahrner et al. 2009). Deletion or mutation of the region (Li et al. 2007; Takahashi et al. 2007; Yuan et al. 2009) has revealed significantly reduced Orai1 Ca^2+^ currents, comparable to those of Orai2 and Orai3 wild-type (Fig. 5B).

Furthermore, STIM1 mediated currents of Orai isoforms differ in FCDI (Lis et al. 2007) (Fig. 5C). While all Orai channels exhibit FCDI within the first 100 ms of a voltage step to negative potentials, its extent is two- to three-fold stronger for Orai2 and Orai3, than for Orai1 (Lee et al. 2009; Lis et al. 2007; Schindl et al. 2009) (Fig. 5C). Subsequently, Orai1 channel currents exhibit a late reactivation phase reaching a plateau after 1500 ms, while Orai2 and Orai3 currents continue to inactivate slowly (Lis et al. 2007; Schindl et al. 2009). These functional differences have been reported to occur due to structural alterations. Reactivation of Orai1 currents has been demonstrated to be controlled by its N-terminal proline/arginine-rich region (Frischauf et al. 2011). Further Orai C-termini control inactivation in a complex manner. The exchange of an isoform-specific C-terminus by that of another Orai homologue can alter fast inactivation (Lee et al. 2009). More importantly, the cytosolic regions (N-, C-terminus and intracellular loop) of Orai channels control FCDI in a cooperative manner (Frischauf et al. 2011). Native CRAC currents show more pronounced inactivation and the reactivation observed for Orai1 is lacking. A co-expression of Orai1 and Orai3, suggested to lead to the formation of heteromeric Orai channels, has shown enhanced FCDI compared to Orai1, but reduced FCDI compared to Orai3 homomeric channels. This suggests that in contrast to ectopically expressed Orai proteins, inactivation of native CRAC channels (Derler et al. 2009b; Zweifach and Lewis 1995a) is obviously controlled by further components.

Another common CRAC channel characteristic, demonstrated by the increase in *I*_DVF_ compared to *I*_Ca2+_, occurs for all Orai isoforms (Fig. 5D). However, interestingly their currents differ in the ratio of *I*_DVF_ versus *I*_Ca2+_. For CRAC channels, it has been hypothesized that this prominent enhancement in current densities corresponds to the extent of inactivation (Prakriya and Lewis 2006). Indeed, Orai3 channels with a more pronounced FCDI than Orai1 exhibit enhanced *I*_DVF_ versus *I*_Ca2+_ than Orai1 (Fig. 5D).

Maintenance of store-operated activation of Orai channels requires part of the ETON region in an isoform-specific manner. While Orai1 requires the last two-third (Orai1 aa 76–90), Orai3 requires only the second-half (Orai3 aa 55–65) of the ETON region for preserved activation by STIM1 (Fig. 5A). This isoform-specific regulation underlies an altered conformation of the cytosolic loop2 regions in the two Orai isoforms. Indeed, an Orai1–Orai3–loop2 chimera remains functional upon N-terminal truncations to aa Y80 (aa Y55 in Orai3), in contrast to wild-type Orai1, which already loses function upon deletion till aa W76 (aa W51 in Orai3) (Fig. 5A). As explained in detail in “Essential coupling domains within Orai1” only loop2 of Orai1, but not that of Orai3 forms inhibitory interactions with the N-terminus of Orai1. A release of those inhibitory interactions can recover Orai channel activation.

Moreover, CRAC channel hallmarks are adjusted by the ETON region in an isoform-specific manner, which has been shown both, in wild-type and constitutively active Orai mutants. While Orai1 requires the whole ETON region (aa 73–90 in Orai1) to preserve FCDI and *I*_DVF_:*I*_Ca2+_ > 1, Orai3 requires in addition to ETON region one residue upstream (aa 47–65 in Orai3 corresponds to aa 72–90 in Orai1) (Fig. 5A). These differences are triggered by a different communication of the N-terminus and loop2 of the two isoforms. Chimeric studies further uncovered that the non-conserved residue Q72 (in Orai1, R47 in Orai3) can influence the maintenance of CRAC channel hallmarks. While a deletion mutant Orai1 Δ*N*_1–71_ shows typical CRAC channel characteristics, they are lost for the chimera Orai1 Δ*N*_1–71_ Orai3–L2. Only upon an additional point mutation Q72R within this chimera, typical CRAC channel characteristics were restored. Hence, authentic CRAC channel properties are maintained by an isoform-specific communication of the N-terminus and the loop2.

Additionally, Orai homologues differ in their sensitivities to 2-aminoethyldiphenyl borate (2-APB), a well-known drug affecting function of diverse ion channels and one of the best characterized modulators of CRAC/Orai currents (Derler et al. 2008; Lis et al. 2007; Putney 2010). Similar to native CRAC currents and STIM1 mediated Orai1 currents, also STIM1 mediated Orai2 currents are inhibited by 50 µM 2-APB concentrations. In contrast Orai3 currents are exclusively enhanced independent of the presence or absence of STIM1 and exhibited a double rectifying current/voltage relationship (Peinelt et al. 2008). Hence, 2-APB mediated Orai3 currents are in sharp contrast to the store-operated ones. They lack high Ca^2+^ selectivity and exhibit an enlarged pore diameter, which suggests that the chemical compound regulates ion channel selectivity of Orai3 channels (Derler et al. 2013b) (Fig. 5E).

In summary, STIM1 mediated currents of the Orai isoforms exhibit slightly distinct CRAC channel hallmarks, which mainly underlies distinct structural features of the ion channels. Moreover, the Orai isoforms require distinct segments of the fully conserved N-terminal ETON region to fully preserve CRAC channel hallmarks.

### Perspectives

The discovery of the two molecular key players of CRAC channels, STIM1 and Orai1, has paved the way to substantially enhance our understanding on their function, interactions and structure. Gained knowledge provides the basis for the development of novel therapeutical approaches of diseases that are associated with the STIM1 and Orai proteins. Currently, the function and the unique biophysical characteristics of these channels are well understood (Butorac et al. 2018; Derler et al. 2016a). Additionally, milestones have been reached in resolving these proteins at a structural level (Hou et al. 2012, 2018; Stathopulos et al. 2008, 2013; Yang et al. 2012; Zheng et al. 2011). However, despite a huge amount of knowledge on STIM1 and Orai channels has been gained so far, the structure of the entire CRAC channel complex is still missing and the structure–function relationship is only marginally understood. Especially, it still remains to be elucidated how the here-described authentic CRAC channel hallmarks are related to the structure or even dynamic conformational changes of the STIM1/Orai complex. So far, applied technologies lack temporal and spatial precision for a dynamic control of the structure and function when the channel moves from the closed to the open state. Here, light represents an ideal tool to precisely and dynamically control CRAC channel function and downstream signalling processes. Initial attempts have already been taken by several groups to equip STIM1 with light-sensitive modules that allow precise, light-guided activation of CRAC channels (Ishii et al. 2015; Kyung et al. 2015; Ma et al. 2017). Consequently, downstream signalling events can be triggered in a more accurate manner. Moreover, the modulatory role of diverse accessory proteins on CRAC channels enhances the complexity in this Ca^2+^ signalling pathway, and potentially further impacts the typical biophysical characteristics of CRAC channels. Additionally, such proteins provide the possibility for a more fine-tuned interference with potential drugs. Improved understanding of the elaborate CRAC channel machinery enables to engineer, beside classical pore blockers, novel drugs or other tools that interfere with specific steps in the STIM1/Orai activation cascade, and thus are relevant in therapeutic treatments of immune deficiency, autoimmune diseases or allergic disorders.

## Electronic supplementary material

Below is the link to the electronic supplementary material.

**Supplementary Figure** **1: Authentic CRAC channel hallmarks of STIM1/Orai1 currents with the corresponding protocols applied for their detection.** A) Current/voltage (I/V) relationship with a reversal potential (*V*_rev_) > + 50 mV is obtained upon an application of a single voltage ramp applied from − 90 mV to + 90 mV at maximal current activation. B) Time course of currents of a constitutive Orai mutant (for example Orai1 P245L) ± STIM1 (black/red) upon the switch from a Ca^2+^-containing to a Na^+^-containing divalent-free solution (ratio of *I*_DVF_/*I*_Ca2+_ </> 1). Currents are taken at − 74 mV upon repetitive voltage ramps which are applied every 5 s. C) SCDI is detected upon repetitive voltage ramps which are applied every 5 s. D) Examples of FCDI, FCDI followed by reactivation, no FCDI and reactivation in a Ca^2+^-containing solution. The inactivation behaviour is detected upon applied voltage steps from a holding potential of 0 mV to − 70 mV. **Supplementary Figure** **2: Comparison of*I***_**DVF**_**versus*I***_**Ca2+**_ A) of store-operated Ca^2+^ currents of STIM1/Orai1 KO cells expressing STIM1:Orai1 P245L at a ratio of 1:1 or 2:1, of constitutive currents of B) STIM 1 1–474 + Orai1 in comparison to STIM1 + Orai1 C) STIM1–C-terminus (–CT) + Orai1, D) STIM1 233–474 (OASF) + Orai1 and E) STIM1 344–449 (CAD) + Orai1. (DOCX 192 kb)
