# Supplementary figures and images for: Critical parameters maintaining authentic CRAC channel hallmarks
Source: Eur Biophys J. 2019 Mar 21;48(5):425–45. doi: 10.1007/s00249-019-01355-6 (PMC6647248; doi:10.1007/s00249-019-01355-6)

**Supplementary Figure 1**

**
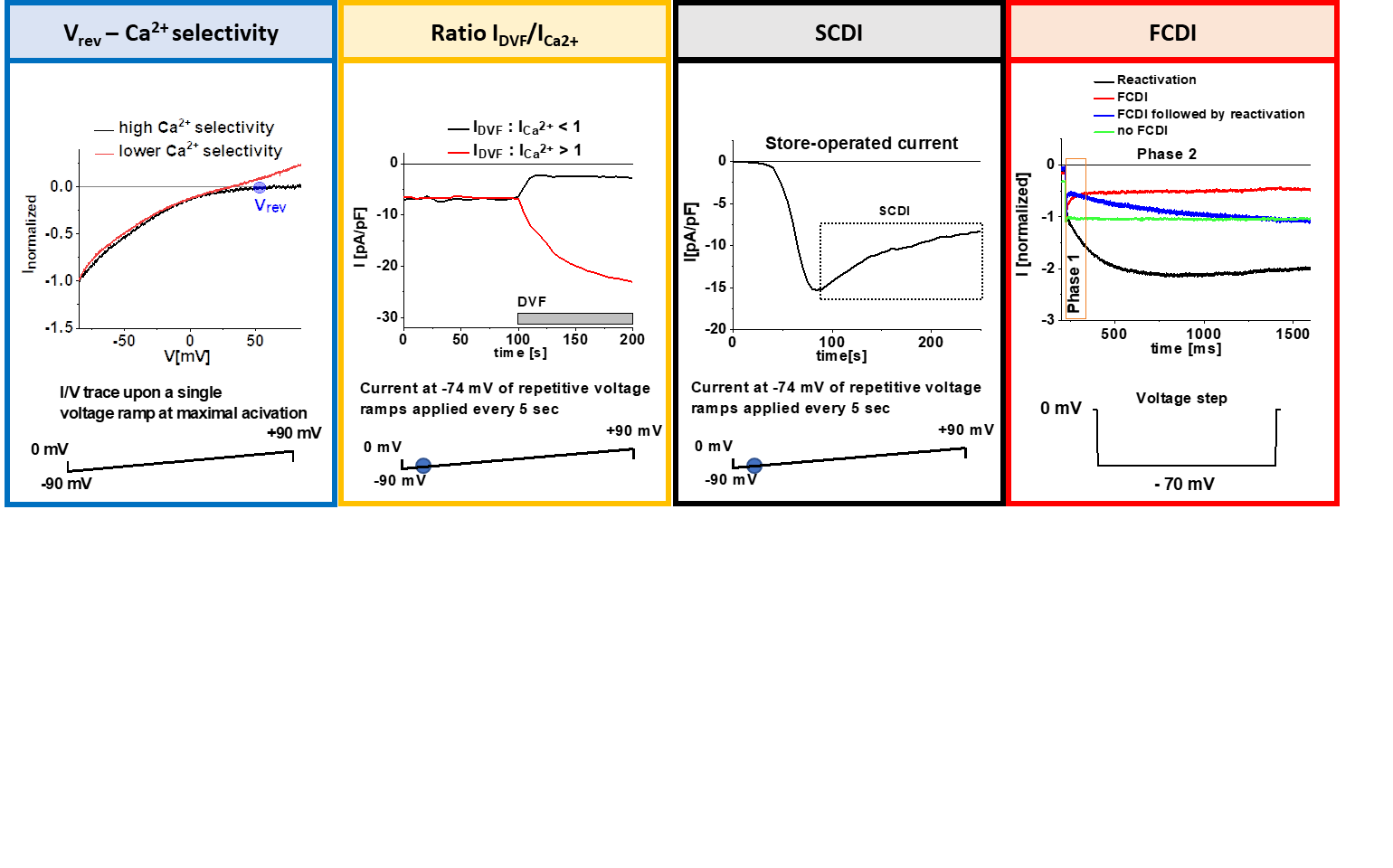
A B C D**

**Supplementary Figure 2**

**A**

**B C**

**D E**

Supplement: Supplementary file 1 — Supplementary Figure 1: Authentic CRAC channel hallmarks of STIM1/Orai1 currents with the corresponding protocols applied for their detection. A) Current/voltage (I/V) relationship with a reversal potential (Vrev) > + 50 mV is obtained upon an application of a single voltage ramp applied from − 90 mV to + 90 mV at maximal current activation. B) Time course of currents of a constitutive Orai mutant (for example Orai1 P245L) ± STIM1 (black/red) upon the switch from a Ca2+-containing to a Na+-containing divalent-free solution (ratio of IDVF/ICa2+ </> 1). Currents are taken at − 74 mV upon repetitive voltage ramps which are applied every 5 s. C) SCDI is detected upon repetitive voltage ramps which are applied every 5 s. D) Examples of FCDI, FCDI followed by reactivation, no FCDI and reactivation in a Ca2+-containing solution. The inactivation behaviour is detected upon applied voltage steps from a holding potential of 0 mV to − 70 mV. Supplementary Figure 2: Comparison ofIDVFversusICa2+ A) of store-operated Ca2+ currents of STIM1/Orai1 KO cells expressing STIM1:Orai1 P245L at a ratio of 1:1 or 2:1, of constitutive currents of B) STIM 1 1–474 + Orai1 in comparison to STIM1 + Orai1 C) STIM1–C-terminus (–CT) + Orai1, D) STIM1 233–474 (OASF) + Orai1 and E) STIM1 344–449 (CAD) + Orai1. (DOCX 192 kb) [file 249_2019_1355_MOESM1_ESM.docx]
